# The impact of rehabilitation sport on breast cancer-related lymphoedema and quality of life

**DOI:** 10.1007/s00404-022-06609-x

**Published:** 2022-07-26

**Authors:** Bettina Boeer, Anna Seller, Birgitt Schoenfisch, Ute krainick-Strobel, Andreas Dietrich, Sara Y. Brucker, Diethelm Wallwiener, Andreas Niess, Markus Hahn

**Affiliations:** 1 Department of Women’s Health, University Hospital of Tuebingen, Calwerstrasse 7, 72076 Tuebingen, Germany; 2 Research Institute for Women’s Health, University of Tuebingen, Tuebingen, Germany; 3 Mammography Screening Centre Tuebingen, Tuebingen, Germany; 4 Faculty of Economics and Social Sciences, Institute for Sports Science, University of Tuebingen, Tuebingen, Germany; 5 Department of Sports Medicine, University Hospital of Tuebingen, Tuebingen, Germany

**Keywords:** Dragon boat, Lymphoedema, Quality of life, Sports, Breast cancer, Rehabilitation

## Abstract

**Purpose:**

Surgery and radiotherapy as part of breast cancer treatment can lead to lymphoedema of the upper extremities (breast cancer-related lymphoedema = BCRL) and reduce the quality of life (health-related quality of life = HRQoL). The aim of the present study was to investigate the influence of paddling in a dragon boat (PP) on HRQoL and BCRL in breast cancer survivors (BCS).

**Methods:**

Between April and October 2017, a prospective case–control study evaluated the effects of PP compared to a control group. In the paddle group (*n* = 28), weekly arm circumference measurements were taken at four defined anatomic areas of the arm before and after training; in the control group (*n* = 70), the measurements were taken once a month. At the beginning and end of the study, questionnaires from both groups (SF 36, EORTC QLQ C30) were evaluated to understand the differences in HRQoL.

**Results:**

The paddle group started with a higher HRQoL compared to the control group. Most interesting, whether the affected or unaffected arm, whether before or after training—the arm circumference decreased over time in the paddling group. A pre-existing lymphoedema was not negatively influenced by paddling. In the paddle group, the physical health was constant over the season, while the physical health of the control group decreased significantly over time.

**Conclusion:**

PP in a dragon boat does not lead to the development or worsening of pre-existing lymphoedema due to breast cancer therapy, and seems to have a positive effect on the quality of life.

## Introduction

Surgery and radiotherapy as part of breast cancer treatment can lead to lymphoedema of the upper extremities (breast cancer-related lymphoedema = BCRL), as well as functional and sensory limitations in the ipsilateral arm. The incidence of lymphoedema is reported between 5 and more than 50% [1]. Quality of life may be restricted by axillary lymphonodectomy and may require lifelong physiotherapy.

Contrary to previous assumptions, several studies have shown that regular physical activity does not carry an increased risk of developing lymphoedema [2–9], and that physical activity has a positive effect on quality of life and overall survival [10, 11].

Paddling in a dragon boat as a rehabilitation sport is known worldwide under the name of "Pink Paddling" (PP) and was initiated by the Canadian sports physician Dr. Donald McKenzie in Vancouver [12] in 1996. There are now 19 official clubs in Germany offering Pink Paddling [13].

There are numerous reports in the literature on the positive effects of the PP on breast cancer survivors (BCS) [7, 12, 14–21], but there was a lack of prospective comparative study on this subject at the time the present study was recruited.

The aim of this prospective study was to evaluate to what extent PP influences health-related quality of life (HRQoL) and the BCRL compared to a control group without regular exercise.

### Materials and methods

The study was a prospective, comparative, longitudinally oriented case–control study conducted from the end of April to the end of October 2017 at the University Breast Centre in Tuebingen (Ethics Committee 014/2015BO2). Inclusion criteria were a completed stage-appropriate therapy with surgery at least 1 year to a maximum of 20 years ago. A signed declaration of consent, a minimum age of 18 with a maximum age of 80 years, and no distant metastases were required. Exclusion criteria for the control group were regular exercise and for the intervention group any contraindications to regular exercise.

#### Study structure

Health-related quality of life was recorded using the SF 36 and EORTC QLQ C30 questionnaires at the beginning and before the end of the study.

The BCS of the paddling group (PG) trained on the local river (Neckar) in the dragon boat under the instruction of a specialist trainer. There were three training groups of eight-to-ten paddlers each; training was carried out once a week for 1.5 h with 1–2 seat changes, so that both arms were equally trained.

Before and after the training, the circumference of both arms was measured with a measuring tape without tension at four defined points.

The control group (CG) measured their arm circumference once a month at the same four measuring points on both arms. In addition to the arm measurements, pain in the upper extremities and the spine were queried on a numerical scale (1 = no pain to 10 = most severe pain).

In this study, lymphoedema was defined as a difference in circumference of at least 2 cm between the affected and unaffected arms [22].

#### Questionnaires to measure quality of life

The HRQoL was collected using the disease-specific, internationally standardised and validated EORTC QLQ-C 30 and the health-related SF 36 [23–33]. The questionnaires on disease-related data and paddling were developed at the Department of Women’s Health in Tübingen.

#### Statistical analysis

Data were collected in REDCap (Research Electronic Data Capture) and analysed using R (Version 3.5.1). Patients’ characteristics were given as mean and standard deviation (SD) numbers and percentages, respectively. Differences between the two groups were tested by *t* tests if variables were normally distributed (such as age or BMI), or using Wilcoxon-Mann–Whitney rank tests for other continuous variables. When comparing values of the same individuals at the start and end of the study, paired tests were chosen. In case of nominal data, the Fisher’s exact test was used for a comparison between the two groups.

EORTC QLQ-C 30 (Version 3.0) and SF 36 scores were calculated following the respective manuals [34]. Arm circumference data were checked for plausibility by looking at the values at a certain position over time and comparing values of the affected and non-affected arm. Obvious outliers were deleted. Since there are measured values for each subject at different points in time, on the affected and non-affected arm and, in case of paddlers, before and after training, a linear multiple regression model with individual as random factor was formulated for evaluation for each arm position. The comparison of paddlers and controls also considered the factors of radiotherapy to the breast and axillary lymph-node removal. Quality-of-life model was assessed using pseudo R^2^ values (according to Nakagawa and Schielzeth).

## Results

**Fig. 1:**
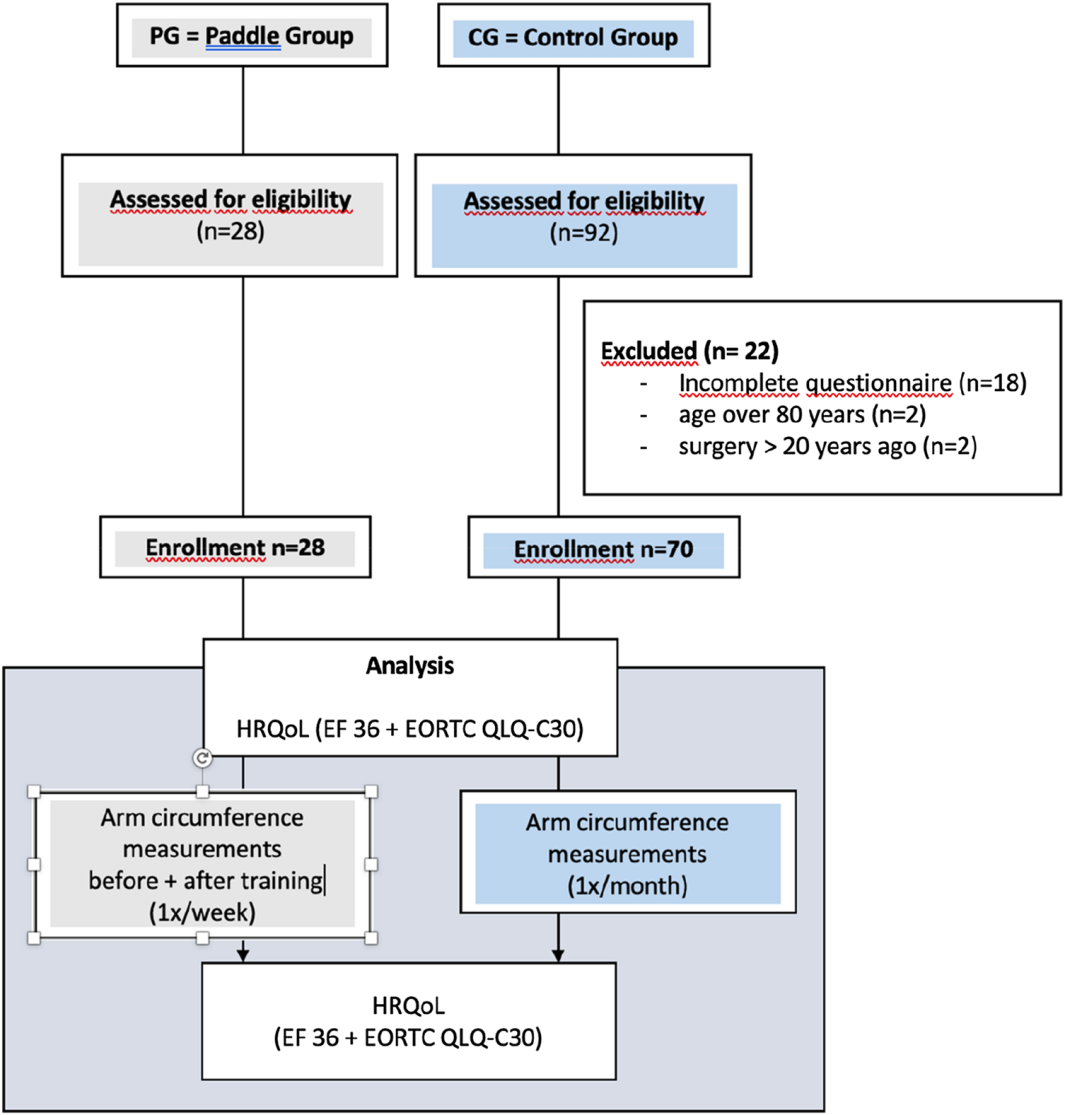
Flowchart: recruitment of study participants + study schedule

Twenty-eight paddlers were screened and included in the PG (paddle group). In the CG (control group), 70 of the 92 screened subjects were included (Fig. 1).

### Patient characteristics

The age at study entry (*p* = 0.254) and the time between last surgery and study entry (*p* = 0.082) did not differ significantly between the two groups. In terms of average BMI alone, the body weight was significantly higher in the control group (mean 24.2 kg/m^2^ with SD 3.1 versus 26.3 kg/m^2^ with SD 4.7, *p* = 0.010) (Table 1). Further data on previous therapies are listed in Table 1.

**Table 1:** Subject characteristics

	Paddle group number respectively mean (SD)	Control group number respectively mean (SD)	*p* Value
BCS	28	70	–
Gender (w/m)	27/1	70/0	0.286^‡^
Age [years]	60.0 (9.2)	62.5 (10.2)	0.254°
BMI [kg/m^2^]	24.2 (3.1)	26.3 (4.7)	0.010°
Time between last surgery and questionnaire [years]	4.7 (4.0)	6.7 (5.1)	0.082°
Final surgery (BCT/mastectomy)	13 / 15	44 / 25	–
SNB	21 (75%)	47 (67%)	0.480^‡^
Prim/secondary AXLND (> 3LN)	18 (64%)	44 (63%)	1.000^‡^
Adjuvant radiotherapy breast/thoracic wall	22 (79%)	59 (84%)	0.559^‡^
Adjuvant radiotherapy LDS	14 (50%)	24 (34%)	0.173^‡^
Adjuvant endocrine therapy	22 (79%)	53 (76%)	1.000^‡^
Adjuvant chemotherapy	15 (54%)	36 (51%)	1.000^‡^
Lymphoedema at study begin	6 (21%)	17 (24%)	1.000^‡^
Lymphoedema at study end	1 (4%)	11 (16%)	0.170^‡^
Pain in affected arm at study begin, [scale 1–10]	1.4 (0.9)	2.8 (2.1)	0.001^†^
Back pain at study begin [scale 1–10]	2.1 (1.6)	3.7 (2.4)	0.001^†^
Tightness due to surgical scar at study begin	1.6 (1.0)	3.0 (2.2)	0.003^†^

°*t* test

^†^ Wilcoxon–Mann–Whitney rank test

^‡^ Fisher’s exact test

### Lymphoedema and pain

A total of 21% (6/28) of the paddlers had lymphoedema—either on the upper or lower arm—at the beginning of the study. By the end of the study, the frequency was only 4% (1/28). In the controls, 25% (17/69) of the participants had lymphoedema at the beginning of the study—mostly on the upper or lower arm, but by the end of the study, the figure had dropped to 16% (11/69). Neither the differences in the proportions of the control or paddle group at the two points in time nor the differences between the groups are significant (all *p* > 0.100).

At the beginning of the study, the paddlers reported significantly less pain in the affected arm and back than the controls (both *p* = 0.001). The feeling of tightness due to the surgical scar was also more pronounced in the controls (*p* = 0.003) (Table 1).

### Evaluation of the questionnaires on quality of life (HRQoL)

#### EORTC QLQ C30

The global health scale among paddlers significantly improved from the beginning of the study to the end of the study (*p* = 0.013). In the control group, there was a significant worsening of the dyspnoea symptom scale (*p* = 0.035).

At the beginning of the study, the controls suffered significantly more often from fatigue (*p *= 0.003) than the paddlers and had significantly poorer role functioning skills (*p* = 0.031).

The paddlers also tended to achieve better scores in the other subscales of the EORTC QLQ C30, but the differences were not significant (Table 2).

**Table 2:** Significance tests for the EORTC

EORTC QLQ C30	*p* Values		
	PG study begin to end (paired test)	CG study begin to end (paired test)	Study begin PG to CG (unpaired test)
QL			
Global health status/QoL	0.013	0.640	0.494
PF			
Physical functioning	0.063	0.137	0.099
RF			
Role functioning	0.888	0.256	0.031*
EF			
Emotional functioning	0.094	0.464	0.424
CF			
Cognitive functioning	0.085	0.409	0.949
SF			
Social functioning	0.528	0.189	0.261
FA			
Fatigue	0.939	0.099	0.003**
NV			
Nausea and vomiting	1.000	0.548	0.856
PA			
Pain	1.000	0.257	0.088
DY			
Dyspnoea	0.572	0.035*	0.234
SL			
Insomnia	0.627	0.556	0.151
AP			
Appetite loss	0.586	1.000	0.398
CO			
Constipation	1.000	0.244	0.249
DI			
Diarrhoea	0.773	0.205	0.104
FI			
Financial difficulties	0.120	0.510	0.992

* significant on 5% level

** significant on 1% level

#### SF 36

At the beginning of the study, the physical summary scale values from the paddle group SF 36 were significantly higher than the values from the controls (*p* = 0.029).

In the control group, the median values at the end of the study were significantly lower than at the beginning (*p* = 0.045). In the paddle group, there was no significant difference in the physical summary scale values at the beginning and the end of the study (*p* = 0.812).

**Fig. 2:**
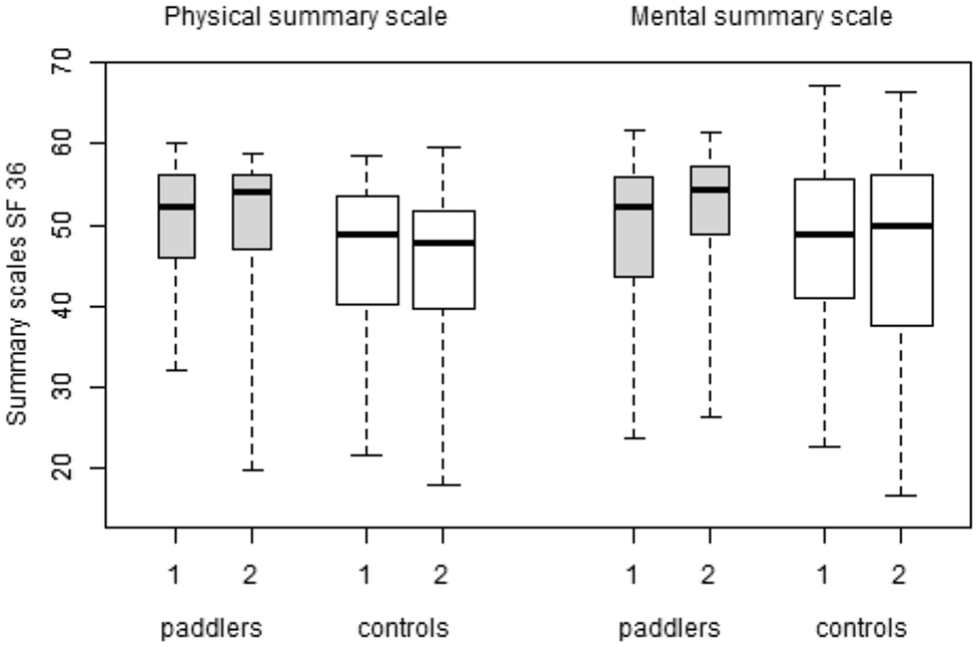
Physical and mental summary scales of SF 36

The mental summary scale values of the SF 36 increased slightly among paddlers from the beginning to the end of the study (as shown in Fig. 2); however, the difference was not significant (*p* = 0.220). The values in the control group did not differ significantly over time (*p* = 1.000), nor did the values at the beginning of the study between the two groups (*p* = 0.861).

### Arm circumference measurements

In the PG, the arm circumference in the regression model decreased significantly both during the training season and after training in all four positions measured (all *p* < 0.001). For three of the four measuring points, significantly smaller circumferences were observed on the affected arm than on the unaffected arm (Table 3).

**Table 3:** Linear regression model with random effects for arm circumference (PG)

Position	Feature	Estimate	*p* Value	Pseudo R^2^
Upper arm	(Intercept)	67.907	< 0.001	0.91
	Affected to unaffected arm	0.059	0.077	
	After to before training	− 0.242	< 0.001	
	Time [per day]	− 0.002	< 0.001	
Lower arm	(Intercept)	98.227	< 0.001	0.88
	Affected to unaffected arm	− 0.206	< 0.001	
	After to before training	− 0.159	< 0.001	
	Time [per day]	− 0.004	< 0.001	
Wrist	(Intercept)	63.629	< 0.001	0.85
	Affected to unaffected arm	− 0.008	< 0.001	
	After to before training	− 0.085	< 0.001	
	Time [per day]	− 0.003	< 0.001	
Hand	(Intercept)	104.437	< 0.001	0.82
	Affected to unaffected arm	− 0.083	< 0.001	
	After to before training	− 0.137	< 0.001	
	Time [per day]	− 0.005	< 0.001	

In the corresponding control model, arm circumference on the upper arm and metacarpus decreased significantly over time (*p* < 0.001 and *p* = 0.045, respectively); in addition, on the upper arm, the affected arm was significantly larger in circumference than the non-affected arm (*p* < 0.001); in the other positions, the difference was not significant (data not shown).

Looking at the paddlers at the beginning of the study and the controls together, a significant reduction in arm circumference is confirmed in all four positions. In women with axillary lymph-node removal (AXLND ≥ 3LN), the measured circumference was larger at all positions, but significantly higher values were found only at the metacarpus (*p* = 0.024). In the present study, radiotherapy showed no significant influence on the arm circumference (Table 4).

**Table 4:** Linear regression model with random effects for arm circumference (PG and CG)

Position	Feature	Estimate	*p*-value	Pseudo R^2^
Upper arm	(Intercept)	63.348	< 0.001	0.90
	Paddler to control	− 0.360	0.522	
	Affected to unaffected arm	0.186	< 0.001	
	Radiotherapy to breast/thoracic wall ± LDS	0.866	0.201	
	AXLND (> 3LN)	0.725	0.173	
	Time [days]	− 0.002	< 0.001	
Lower arm	(Intercept)	65.990	< 0.001	0.87
	Paddler to control	− 0.527	0.193	
	Affected to unaffected arm	− 0.077	0.022	
	Radiotherapy to breast/thoracic wall ± LDS	0.917	0.061	
	AXLND (> 3LN)	0.405	0.287	
	Time [days]	− 0.002	< 0.001	
Wrist	(Intercept)	45.235	< 0.001	0.90
	Paddler to control	− 0.363	0.237	
	Affected to unaffected arm	0.003	0.873	
	Radiotherapy to breast/thoracic wall ± LDS	0.278	0.449	
	AXLND (> 3LN)	0.348	0.231	
	Time [days]	− 0.002	< 0.001	
Hand	(Intercept)	70.396	< 0.001	0.76
	Paddler to control	− 0.007	0.970	
	Affected to unaffected arm	− 0.056	0.019	
	Radiotherapy to breast/thoracic wall ± LDS	− 0.264	0.247	
	AXLND (> 3LN)	0.409	0.024	
	Time [days]	− 0.003	< 0.001	

## Discussion

The aim of this prospective case–control study was to clarify whether pink paddling influences the quality of life and any lymphoedema—quantified by arm circumference measurements.

### Discussion of patient characteristics

Apart from the BMI—which was significantly higher in the controls—there were no significant differences regarding the medical history and personal data of the PG and CG. The fact that the average participant in the CG was overweight (average BMI = 26.3 kg/m^2^) may be due to their general lack of interest in exercise. Most interestingly, the literature describes that an increased BMI is associated with an increased risk of developing postoperative lymphoedema [1, 35–38].

This could also explain the possibly slightly (not significantly) higher frequency of lymphoedema in the control group (25% controls vs. 21% patients at T1; 16% controls vs. 4% patients at T2).

As in the publication by Brown et al. 2004 [22], lymphoedema was defined in the present study as a difference in arm circumference between affected and non-affected arm of at least 2 cm. According to this definition, a seasonal decrease in lymphoedema could be observed over time, which was more pronounced in the PG than in the CG (17 versus 9% difference).

At the beginning of the study, the paddlers reported significantly less pain in the affected arm and back as well as a significantly lower feeling of tightness from the surgical scar than the controls. The CG therefore suffered from subjectively more symptoms than the PG.

Reis et al. [39] were able to confirm in 2018 the effects of physical activity on pain in BCS observed in this study. For example, patients who exercised three times a week had significantly lower absolute pain levels and intensities and significantly lower levels of impairment due to pain in everyday life than the control group without physical activity. The mobility of the arms as well as the strength of both arms increased significantly in the sports group—in contrast to the control group.

### Discussion of the quality of life

One of the major limitations of the study is that the patients were not randomly assigned to each group. The paddle group was pre-existing. Before the start of the paddling season, they had no training within 6 months prior to the measurements (during wintertime). Therefore, the effects of both groups are comparable.

At the beginning, the PG started with a significantly better physical health status (SF 36, *p* = 0.029)—in contrast to the CG—and maintained it over the entire season. The physical health of the CG significantly decreased over time (SF36, *p* = 0.045). This could be due to the overall lower level of physical activity and the lack of opportunities to increase performance over time. Kendall et al. [40] found similar conclusion: patients who were physically active after being diagnosed with cancer achieved a higher quality of life on the physical cumulative scale in SF36.

Regarding the mental summary scale of the SF36, there were no significant differences between the study group.

This also corresponds with the available literature [41, 42]: with increasing disease-free survival, the quality of life of breast cancer patients approaches that of the healthy reference population, independent of interventions or rehabilitation measures.

In the evaluation of the EORTC QOL C30, the results also indicate that no improvement of HRQoL can be achieved without sports intervention. Thus, only the PG achieved a significant increase in the subscale of global health.

Role functioning was already significantly higher in the PG than in the CG at the beginning of the study. This could be explained by participation in team sports with the assumption of certain roles and functions within the group that existed before the study began.

The present study showed that the CG suffered significantly more from fatigue than the PG. This result has already been shown by Kessels et al. [43] for physical activity in general and Ray et al. [18] specifically for dragon boat paddling: regular physical activity has a positive effect on cancer-associated fatigue in cancer survivors.

Iacorossi et al. [44] were also able to determine this result in a similar setting through a singular evaluation of EORTC: the PP achieved a better HRQoL in the “operational and symptoms areas” than the CG, but the authors did not mention the time of measurement in their publication.

Overall, it can be stated that the developments in HRQoL identified in this study and the results of EORTC QLQ C30 and SF 36 fit into the existing knowledge of the effects of physical activity on BCS.

### Discussion of arm circumference measurements

Whether the affected or unaffected arm, whether before or after training—the measured arm circumference decreased significantly in the PG over time. Thus, no negative effect of dragon boat training on a possibly pre-existing BCRL could be observed.

Iacorossi et al. [44] came to a similar conclusion regarding the influence of PP on lymphoedema incidence. In the Italian study, measurements of the arms were taken once at the beginning and end of the study. In contrast to the present study, neither the progression over the training season nor possible differences due to individual training sessions were recorded. Looking at the two time points, a decrease in lymphoedema was observed in the Italian paddling group at the end of the study, as arm circumferences had decreased at almost all measurement points by the end of the study.

The decrease in arm circumference, also visible in the control group but less pronounced, could also be due to muscle atrophy caused by increased rest.

The fact that the number of removed lymph nodes has a clear influence on the arm circumference is shown both in the present study and in the literature. In women with AXLND, the measured arm circumference was greater in all positions, but significance was only achieved in the middle hand (*p* = 0.024).

Haid et al. [45] were able to show in their publication that patients with AXLND suffer significantly more frequently from restricted movement, lymphoedema, and loss of sensitivity compared to SNLB alone. Helms et al. [46], Tsai et al. [47], Kilbreath et al. [48], and Bromham et al. came to similar conclusions in their 2017 review [49].

In the present study, adjuvant radiotherapy was not a significant factor in the development of BCRL. However, many recent studies show that radiotherapy to the breast and thoracic wall and lymphatic drainage system are relevant risk factors for the development of arm lymphoedema [38, 47, 48, 50].

## Conclusions

Rehabilitation sport in dragon boat does not lead to the development or aggravation of pre-existing lymphoedema after breast cancer therapy and is therefore a useful rehabilitation sport with a positive effect on the quality of life.

We conclude that from a medical point of view, exercise should be recommended as part of the overall treatment plan for all patients with breast cancer who have no contraindication to exercise. Pink Paddling is well suited as a rehabilitation sport after breast cancer.
